# Differences of oribatid mite community and trophic structure between karst caves and surface different moss habitats

**DOI:** 10.1371/journal.pone.0290144

**Published:** 2023-08-17

**Authors:** Tong Gong, Xiumei Yin, Wenjun Liu, Hu Chen, Zheng Shi

**Affiliations:** School of Karst Science, Guizhou Normal University, Guiyang, China; East Carolina University, UNITED STATES

## Abstract

In order to reveal the community characteristics and trophic structure of oribatid mites in different moss habitats in karst caves, the oribatid mites in the moss habitats of ground (GD), understory (US), cave wall (CW), surface shrub (SB) and farmland (FL) outside the cave were collected in October 2021. Through the identification and data analysis of oribatid mites, 2352 oribatid mites were found, belonging to 45 families and 72 genera, most of which were Gymnonota. The family number, genus number, individual number, individual density, dominant genus composition, community diversity, community similarity, MGP (Analysis methods for ecological groups of oribatid mites) ecological group of oribatid mites and trophic structure of oribatid mites in different moss were analyzed. The results showed that: (1) The number of families, genera, individuals, and individual density of SB and FL are higher than those of the other three habitats; (2) *Platyliodes*, *Oppiella*, *Tectocepheus*, *Scutovertex*, *Scheloribates* and *Trichogalumna* are the dominant genera of the oribatid mites in the cave moss habitat, among them, *Tectocepheus* and *Trichogaluna* have the most obvious advantages; (3) The diversity index of shrub (SB) was higher than that of other four habitats; Similarity between ground and cave wall, shrub and farmland is high; (4) The MGP ecological group of oribatid mites in different habitats is dominated by O type (Overall type belongs to MGP analysis results, 20%≤M,G,P≤50%), and a total of 42 genera of oribatid mites preliminarily constitute the trophic structure of oribatid mites in the cave moss habitat. Based on the above results, it can be concluded that there are differences in the community structure of oribatid mites in different moss habitats in the study area, and the use of dominant genera of mites can preliminarily indicate the environmental conditions of different moss habitats. This study enriches the study of mites in karst cave mosses, and can provide theoretical significance for the protection of cave biodiversity in karst areas.

## 1 Introduction

With the Guizhou Plateau as the center, the karst area in southern China reaches 550000 km^2^, which is the largest and most developed area of tropical and subtropical karst in China and even in the world [1]. Karst environmental and ecological problems are strongly representative in the world. Guizhou is the center of the largest Karst Plateau area in China and even in the world, where a set of the most typical and remarkable Karst caves are developed. The caves are complex in structure and diverse in types. China has a large number of caves and rich cave biological resources [2–4]. The plant types are relatively single [5], with less distribution, and most of them are herbaceous plants such as bryophytes and ferns. Caves are a unique but less studied environment, especially the fauna, mainly arthropods, including oribatid mites. Due to the difficulties in taxonomy and methods related to sampling, the research on acarology in caves has been very scarce [6]. Most cave mites are distributed in different microhabitats. At present, many studies on cave mites are about new species and water mites [7–11].

The oribatid mite is very suitable as an indicator species for the ecological environment, as it exists in most terrestrial habitats, has high diversity and abundance, is easy to sample, exhibits good adaptability to multiple environments, and has low spatial mobility [12], they rarely migrate over long distances [13]. The oribatid mites have different indicating functions. In the Karst Rocky desertification area, the *Trichogalumna* can preliminarily indicate the rocky desertification environment [14]. The oribatid mite is also an important indicator species of past environmental changes, playing an important role in the nutrient cycle of the ecosystem and the formation of Humus [15], It also plays an indispensable role in litter decomposition and trophic cycling [16]. The study of the community structure and trophic structure of oribatid mites plays an important role in various fields such as air pollution control and forest soil succession changes [17], the oribatid mite generally has a complete trophic structure in different environments, and some oribatid mites are predators of nematodes and slow-moving or injured snails [18]. The presence of complex trophic networks in geothermal active lava fields by oribatid mites [19], the mites on the bark of dead trees span at least three trophic levels [20]. Oribatid mites or moss mites dominate moss-dominated Moss-dominated biocrust and provide essential ecosystem services such as decomposition and nutrient cycling [21]. A better understanding of the trophic structure and decomposition function group of oribatid mites will not only help to understand the Species diversity of oribatid mites in the study area, but also have a positive significance to understand the functions of these oribatid mites in the process of organic [22].

Moss as a typical pioneer plant in caves [23], and its attached stone or epiphytic cover is a very suitable model for ecological research on habitats [24], disturbed caves are usually dominated by drought resistant and tough Bryophyte, while relatively undisturbed caves are rich in Bryophyte, which are more adaptable to humid environments [25]. There are a large number of Arthropod communities living in the moss, and the number of oribatid mites is higher [26], however, there is relatively little research on mites in moss. The diversity of cave plants is closely related to habitat heterogeneity, light and water conditions and nutrients. The protection and restoration of Bryophyte in caves can promote the settlement, growth and succession of cave Vascular plant. Bryophyte can be used as an indicator of the overall plant diversity and recovery status of Karst caves [25].

In recent years, the academic research on oribatid mites has been increasing, but the Species diversity of oribatid mites in Karst cave moss habitats still needs further exploration. In this paper, the composition and distribution, diversity, similarity, ecological groups and trophic structure of oribatid mites in different moss habitats of karst caves were studied, providing theoretical basis for the protection of cave biodiversity in karst areas, in recent years, the academic research on oribatid mites has been increasing, but the Species diversity of oribatid mites in Karst cave moss habitats still needs further exploration. In this paper, the composition and distribution, diversity, similarity, ecological groups and trophic structure of oribatid mites in different moss habitats of karst caves were studied, providing theoretical basis for the protection of cave biodiversity in karst areas, the research results also have a certain reference value for the protection and restoration of animal and plant communities in Karst caves, the study of oribatid mites in mosses of different habitats in caves is helpful to understand the response of oribatid mites to special environments, and is of great significance for further understanding of Karst ecological processes. To explore the ecological processes above and below the ground in Karst area and reveal the response mechanism of oribatid mites in special environments.

## 2 Materials and methods

### 2.1 Overview of the study area

**Fig 1:**
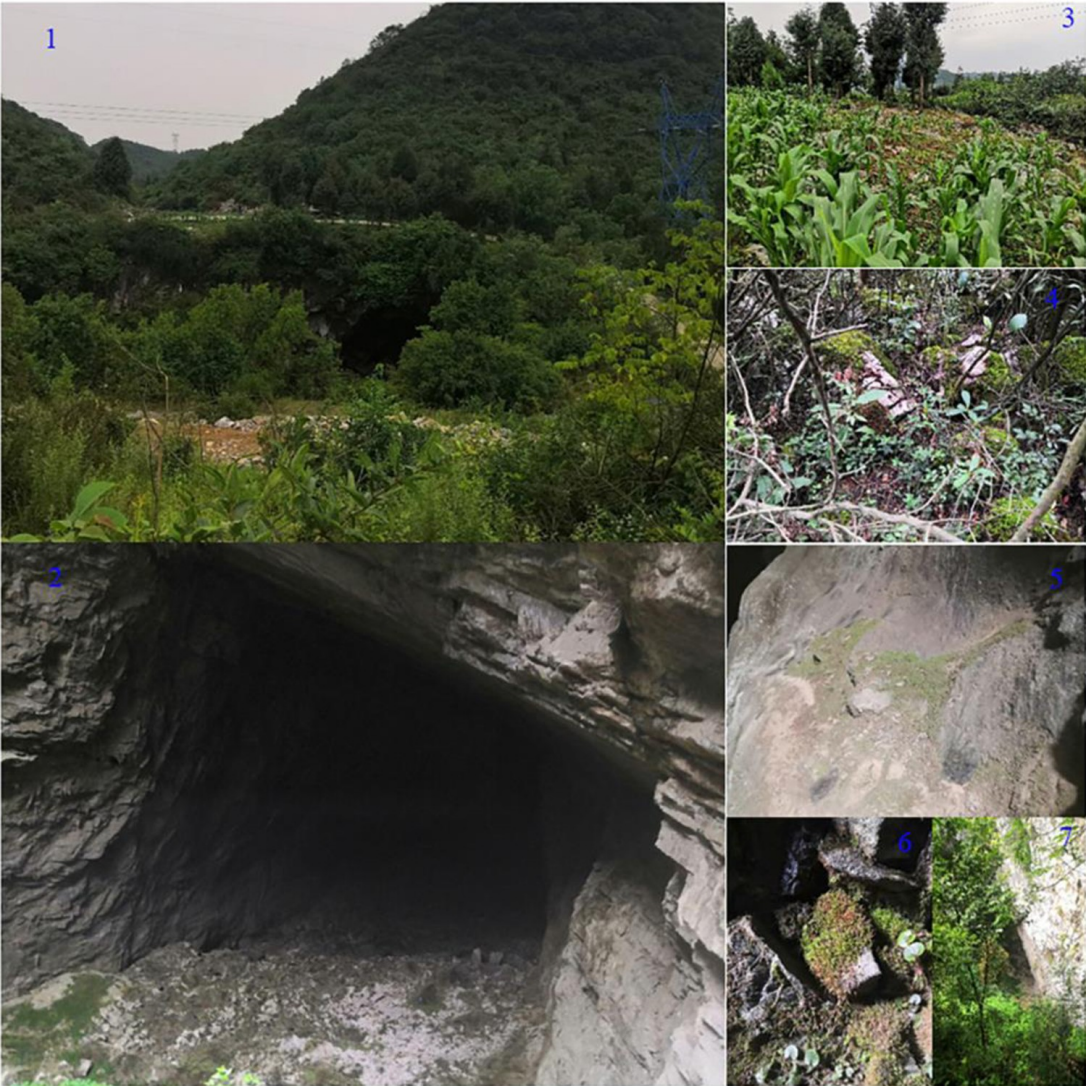
Wangtian Cave portal information and sampling point settings. Note: 1. Surroundings outside the cave; 2. Portal environment; 3. Farmland (FL); 4. shrub (SB); 5. cave wall (CW); 6. ground (GD); 7. understory (US).

The study area is located in Qianxi County, Bijie City, Guizhou Province, southwest China (105° 47 ′ E ~ 106° 26 ′ E, 26° 45 ′ N ~ 27° 21 ′ N). Qianxi County is high in the northwest and low in the southeast. The highest point in the territory is 1679.3m above sea level, the lowest point is 760m above sea level, and the average altitude is 1250m [27]. The climate in the area belongs to the subtropical monsoon climate. The annual average frost free period is 264 days, the annual average temperature is 13°C, and the annual average precipitation is 1000~1170mm. Atmospheric precipitation is the main source of groundwater recharge in the area, and the annual average sunshine hours are 1348.9h. The soils developed in this area mainly include light clay thin layer of black lime soil, light gravelly heavy soil medium thick layer of ordinary yellow soil, light gravelly heavy soil thin layer of acid purple soil. The forest land area of Qianxi City is 947km^2^. The forest coverage rate is 53.91%. The area includes coniferous forests of Pinus massoniana, Pinus yunnanensis, fir, etc. and broad-leaved forests of oak, poplar, paulownia, etc. Food crops mainly include rice, corn, potato, etc. Cash crop mainly include flue-cured tobacco and soil tobacco. Due to the particularity of the geographical location and geological and geomorphic combination of the area, as well as the influence of lithology, structure, and strong downcutting of rivers, caves of different sizes and natures have been formed. The exposed area of carbonate rocks in the study area is large, mostly exposed, and the distribution area is more than 85%. It is a typical karst area. The Wangtian Cave selected in this paper is located in Honglin Yi and Miao Township in the northwest of Qianxi County, with an elevation of 1470m and a height difference of about 100m. It is a typical karst sinkhole cave [28]. The cave plant resources in Wangtian Cave are abundant (Table 1), The vegetation near the entrance of the cave includes *Euphorbia spinosa*, *Pilea pumila*, etc. There are contiguous shrublands and agricultural corn fields outside the cave, and there are understory shrublands at the cave entrance. There are many crushed stones distributed on the ground of the cave entrance. There is a large amount of attached moss on the cave wall, cave entrance ground, and cave entrance understory shrublands, as well as on the shrublands outside the cave and the agricultural land outside the cave. There are few signs of human interference (Fig 1).

**Table 1:** Dominant species of plants at the entrance of Wangtian Cave and outside the cave.

position	dominant species	Vegetation type
Stone ground	*Polystichum acanthophyllum*	herbaceous
	*Begonia henryi*	
East side of the entrance to cave	*Elatostema oblongifolium*	herbaceous
	*Pteris actiniopteroides*	
Entrance to cave	*Salix dunnii*	macrophanerophytes
	*Aesculus chinensis*	
	*Stachyurus himalaicus*	shrub
	*Rubus multibracteatus*	
	*Woodwardia japonica*	herbaceous
	*Coniogramme affinis*	
	*Pilea pumila*	
Shrub outside the cave	*Rhus punjabensis*	shrub
	*Rhus chinensis*	
	*Quercus fabri*	
	*Rosa multiflora*	
	*Corylus heterophylla*	
	*pieris japonica*	
	*Polystichum tsus-simense*	herbaceous

### 2.2 Research methods

#### 2.2.1 Sample plot setting and sample collection

**Fig 2:**
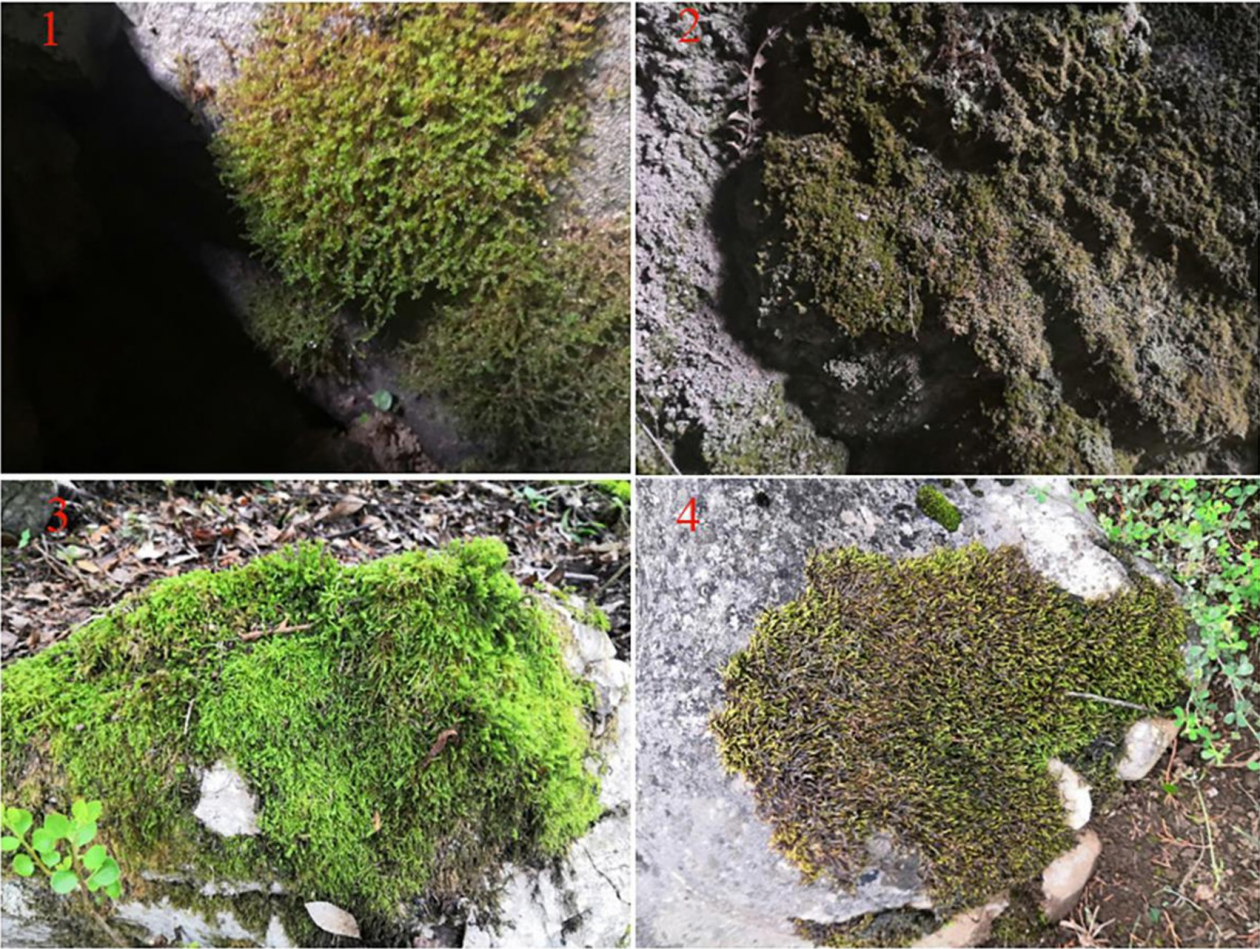
Partial moss in different habitats. Note: 1. Moss on the ground in caves; 2. The moss on the cave wall; 3. Middle moss in the shrub outside the cave; 4. Moss in Farmland Outside the Cave.

In October 2021, a field survey was conducted in Wangtian Cave, the cave habitat is selected within 10 meters outside the entrance of the cave and within 20 meters of the weak light zone inside the cave. The near surface habitat is selected within 10 to 30 meters outside the entrance, including five typical habitats of stony bryophytes were selected, including ground (GD), shrub (SB), understory (US), cave wall (CW) and farmland (FL), in a sequence from outside to inside. All moss is rocky moss. According to the actual conditions of the five habitats, four duplicate plots were set up for each habitat, and a total of 20 plots were set up. Each sample plot is collected at a certain distance according to the actual situation 1 × 10 × 10 cm of moss [29], the collected moss is in the sample area with less human interference, relatively uniform thickness of moss, and relatively flat stones, the dominant species of moss in different habitats are different, and the dominant species of moss in ground, shrubs, understory, cave walls, and farmland are Dicrranodontium filifolium, Bryonoguchia molkenboeri, Brachythecium helminthocladum, Dicranella micro~divariata and Erythrodontium julaceum (Fig 2). All samples are put into cotton bags with good air permeability and numbered before being brought back to the laboratory for analysis. Due to only selecting Wangtian Cave as the research area this time and not selecting more caves, it is hoped that future research can enrich the habitat and cave types.

#### 2.2.2 Collection and identification of oribatid mites

Place all the moss samples in the Tullgren funnel, and bake them continuously for 48 hours with 60W incandescent lamp. During the baking, turn the lamp on and off for 15–20 minutes at intervals. The baking temperature is controlled at about 35°C. Put a beaker with 75% alcohol dilution solution under the Tullgren funnel to collect the moss moving object samples. After that, the transparent specimens were made into temporary slides and placed under the microscope of Olympus (Olympus CX41RF). The collected oribatid mites were identified with reference to the books such as " Pictorical Keys to Soil Animals of China" [30], "Acrology" [31] and "A Manual of Acrology (3rd edition)" [32]. Due to the unclear identification characteristics of nymphs and damaged residual insects, statistics and identification of nymphs and residual insects were not conducted, all specimens were identified to the first order of genus, and the individual number of oribatid mites was counted, at present, our research on oribatid mites can only reach the genus level. The classification order of oribatid mites in this paper is mainly based on "A Manual of Acarology (3rd edition)" [32].

#### 2.2.3 Data analysis

(1) Quantitative dominance [33]: according to Zheng’s method, those whose individual number accounts for more than 10% of the total catch are marked as dominant groups (+++), those whose proportion is 1% - 10% are marked as common groups (++), and those whose proportion is less than 1% are rare groups (+).

(2) Community diversity analysis [34]

$$Shannon\;wiener\;diversity\;index:\;H\prime =-\sum_{i\;=\;1}^{s}P_{i}\;\ln\;P_{i}$$ (1)

Margalefrichnessindex:SR=(S−1)/lnN (2)

$$Pierou\;evenness\;index:\;J=H^{\prime }/\ln\;S$$ (3)

$$Simpson\;dominance\;index:\;C=\sum_{i\;=\;1}^{s}{\left(n_{i}/N\right)}^{2}$$ (4)

Where: *s* is the number of groups, *N* is the total number of mite individuals, *ni* is the number of individuals of group *i*, and *Pi* is the proportion of the number of individuals of group *i* in the total number of individuals of the community.

(3) Analysis of community similarity [35]

Jaccardcommunitysimilaritycoefficient:q=c/(a+b−c) (5)

Where: a is the number of community groups A, b is the number of community groups B, and c is the number of common groups of the two communities. (0 < q < 0.25 is very dissimilar, 0.25 ≤ q < 0.5 is medium dissimilar, 0.5 ≤ q < 0.75 is medium similar, 0.75 ≤ q < 1 is very similar)

(4) MGP analysis of the ecological structure of oribatid mites [36]: MGP analysis was used to classify the mites into 3 groups: M stands for Macrophylla, G stands for Gymnonota, and P stands for Poronota. MGP—Ⅰ analysis was used to calculate the percentage of genus number of M, G and P groups, and MGP—Ⅱ analysis was used to calculate the percentage of individual number of M, G and P groups. See Table 2 for classification criteria.

**Table 2:** Classification criteria of community types on soil mites (Oribatida).

Community type	Abbreviation	Criteria
Macropylina type	M	M>50%
Gymnonota type	G	G>50%
Poronota type	P	P>50%
Overall type	O	20%≤M,G,P≤50%
Macropylina-Gymnonota type	MG	20%≤M,G≤50%,P<20%
Gymnonota-Poronota type	GP	20%≤G,P≤50%,M<20%
Macropylina-Poronota type	MP	20%≤M,P≤50%,G<20%

Note: M, G and P in the division criteria represent the number of groups and genera and the proportion of individuals of M, G and P in MGP-Ⅰ and MGP-Ⅱ analysis respectively.

(5) Data statistics and analysis: data statistics are compiled with Microsoft Excel 2020 software; The data were analyzed by IBM SPSS 22.0 software. The single factor analysis of variance (ANOVA) was used to analyze the community differences. The significance level was p<0.05; Data mapping is carried out in Origin 2021 and IBM SPSS 22.0 software.

## 3 Results and analysis

### 3.1 Composition and distribution characteristics of oribatid mite community in different moss habitats

2352 oribatid mites were captured in different moss habitats in the study area, belonging to 45 families and 72 genera (S1 Appendix). Among them, there are 255 members in 24 genera of 14 families in Group M (Macrophylla), 1150 members in 27 genera of 18 families in Group G (Gymnonota) (*Tectocepheus* occupies the absolute advantage), and 947 members in 21 genera of 13 families in Group P (Poronota) (the group and quantity of oribatid mites are evenly distributed in different habitats). The composition and quantity distribution of oribatid mites community in the five moss are shown in S1 Appendix. 211 oribatid mites in 15 families, 21 genera, are captured on the ground (GD), 773 oribatid mites in 39 families, 60 genera, 39 families in the shrub (SB), 118 oribatid mites in 25 genera, 21 families in the understory (US), 63 oribatid mites in 16 genera, 15 families in the cave wall (CW), and 1187 oribatid mites in 31 families, 41 genera, 31 families in the farmland (FL). It can be seen that Gymnodota and Poronota are in the majority in the five moss habitats. There are abundant group and individual numbers of oribatid mites in the mosses of shrub and farmland, while the group and individual numbers of oribatid mites in the ground and cave wall are relatively small.

**Fig 3:**
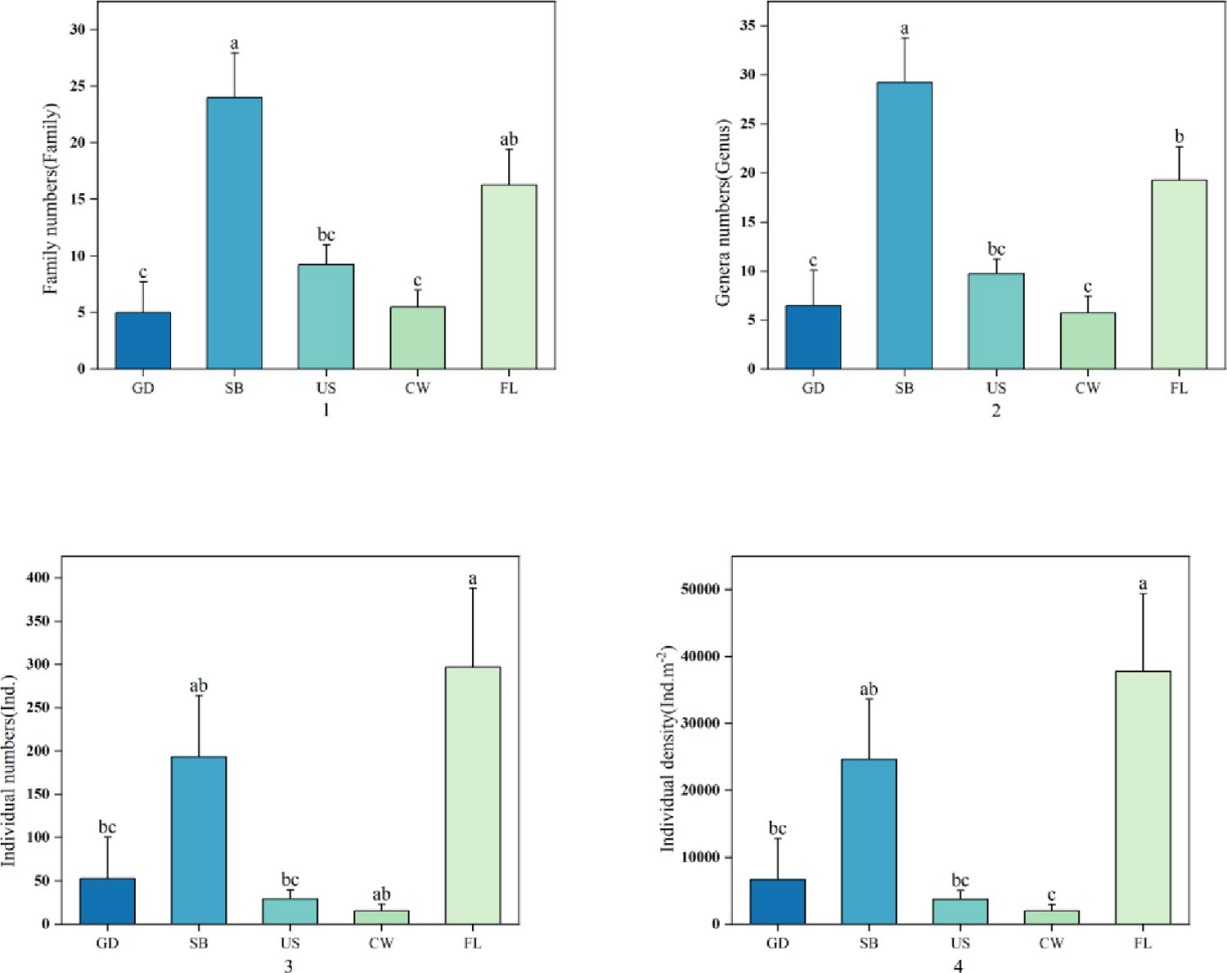
Distribution of family number (1) and genus number (2), individual number (3) and individual density (4) of Oribatid mites in different moss habitats. Note: Different lowercase letters indicate significant differences in the distribution of family number, genus number, individual number and individual density in different habitats. Ground (GD), understory (US), cave wall (CW), surface shrub (SB) and farmland (FL).

**Fig 4:**
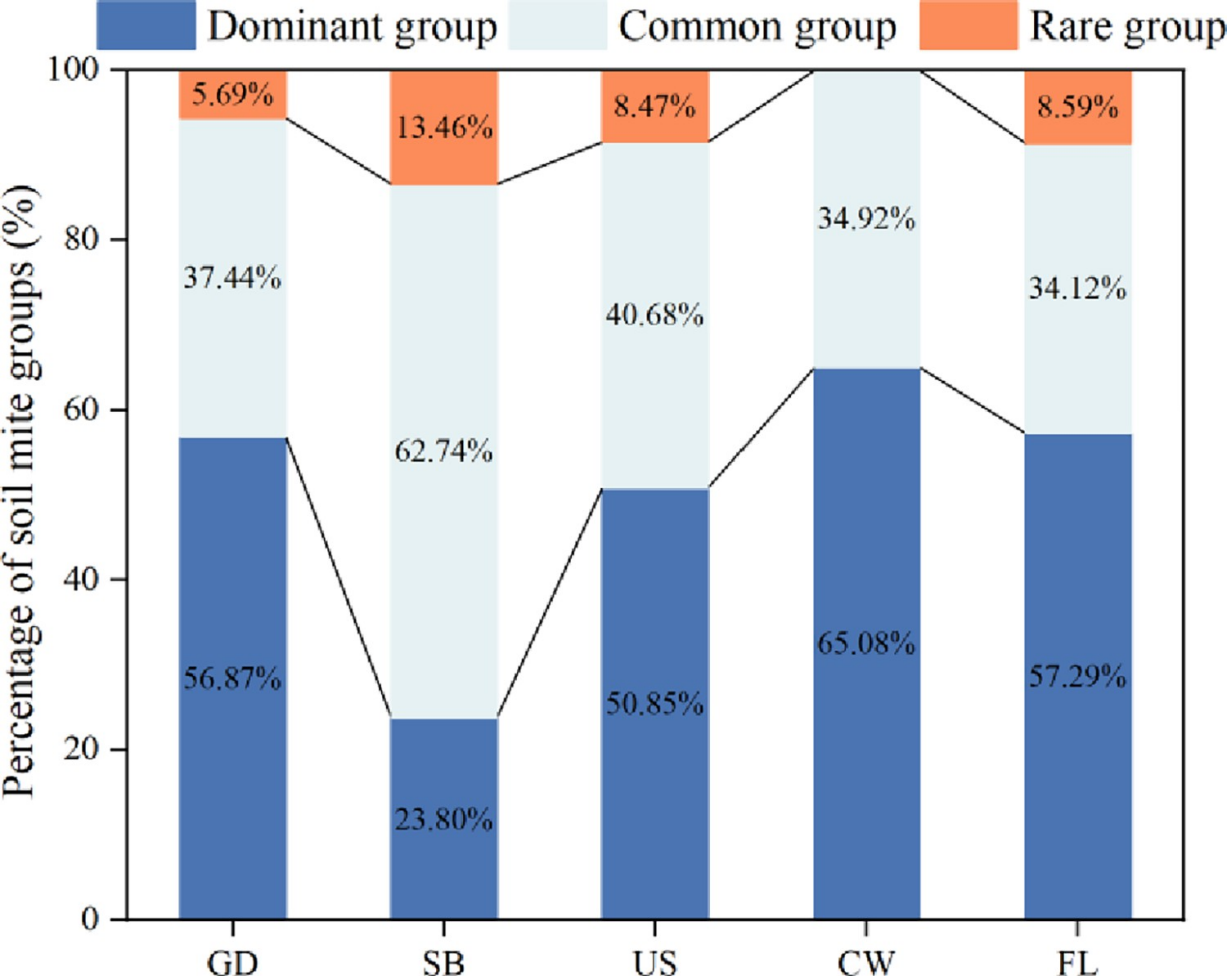
Composition of dominant genera of oribatid mites in different moss habitats. Note: Ground (GD), understory (US), cave wall (CW), surface shrub (SB) and farmland (FL).

At the family level of oribatid mites (Fig 3–1), the number of families of shrub is the largest, and the number of families on the ground and cave wall is the smallest, which is shown as SB>FL>US>GD, CW. There is a significant difference between the ground, cave wall and shrub (F = 8.498, *df* = 4, p<0.05). There is a significant difference between the ground, cave wall and farmland (F = 8.498, *df* = 4, p<0.05). There is a significant difference between the shrub, ground, understory and cave wall (F = 8.498, *df* = 4, p<0.05), and there is no significant difference between other habitats (F = 8.498, *df* = 4, p>0.05); At the genus level of oribatid mites (Fig 3–2), the number of oribatid mites in the moss habitats of shrub is the largest, and the number of oribatid mites in the moss habitats of ground is the smallest, which is SB>FL>US>CW>GD. Among them, there is a significant difference between the ground, cave wall and shrub (F = 10.028, *df* = 4, p<0.05), there is a significant difference between the ground, cave wall and farmland (F = 10.028, *df* = 4, p<0.05), there is a significant difference between the shrub and the other four habitats (F = 10.028, *df* = 4, p<0.05), and there is no significant difference between other habitats (F = 10.028, *df* = 4, p>0.05); In the individual number of oribatid mites (Fig 3–3), the number of oribatid mites in farmland is the most abundant, and the number of oribatid mites in cave wall is the least, in the order of FL>SB>GD>US>CW. Among them, there were significant differences among ground, understory and farmland (F = 4.759, *df* = 4, p < 0.05), significant differences among shrub and cave wall (F = 4.759, *df* = 4, p < 0.05), significant differences among shrub, farmland and cave wall (F = 4.759, *df* = 4, p < 0.05), but no significant differences among other habitats (F = 4.759, *df* = 4, p > 0.05). The variation of the individual density of oribatid mites and their differences are consistent with the variation of the individual number (Fig 3–4) The individual density of oribatid mites in farmland is the highest, and that in cave wall is the lowest, showing FL>SB>GD>US>CW. It can be seen that the number of families, genera, individuals and density of oribatid mites change with the change of moss habitat.

From the composition of dominant genera, common genera and rare genera of oribatid mites, it can be found that the composition of oribatid mites in the five moss habitats shows a large difference (Fig 4). In the ground habitat, the dominant genera of oribatid mites are *Oppiella*, *Tectocepeus* and *Scutovertex*, and the rare genera are *Hypochthonius*, *Dolicheemaeus* and *Xylobates*. The number of individuals of dominant, common and rare genera respectively accounts for 56.87%, 37.44% and 5.69% of the total number of individuals in the habitat. In the shrub habitat, the dominant genus of oribatid mites is *Palatyloides*, and the rare genera are *Lepidacarus*, *Cepheus* and *Incabates*. The number of individuals of dominant, common and rare genera respectively accounts for 23.80%, 62.74% and 13.46% of the total number of individuals in the habitat. In the understory habitat, the dominant genera of oribatid mites are *Tectocepeus*, *Scutovertex* and *Trichogalumna*, and the rare genera are *Lepidozetes*, *Phyllhermania* and *Oribatella*. The number of individuals of dominant, common and rare genera respectively accounts for 50.85%, 40.68% and 8.47% of the total number of individuals in the habitat. In the cave wall habitat, the dominant genera of oribatid mites are *Tectocepeus*, *Scutovertex* and *Scheloribates*, and the number of individuals of dominant, common and rare genera respectively accounts for 65.08%, 34.92% and 0.00% of the total number of individuals in the habitat. In the farmland habitat, the dominant genera of oribatid mites are *Tectocepheus* and *Trichogaluna*, and the rare genera are *Protorobotritia*, *Yoshibodes* and *Podoribates*. The number of individuals of dominant, common and rare genera respectively accounts for 57.29%, 34.12% and 8.59% of the total number of individuals in the habitat. It can be seen that there are some differences in the dominant genera of oribatid mites distributed in different moss, and the individual numbers of *Tectocepheus* and *Trichogalumna* occupy an absolute dominant position.

### 3.2 Diversity and similarity analysis of oribatid mite community in different moss habitats

**Fig 5:**
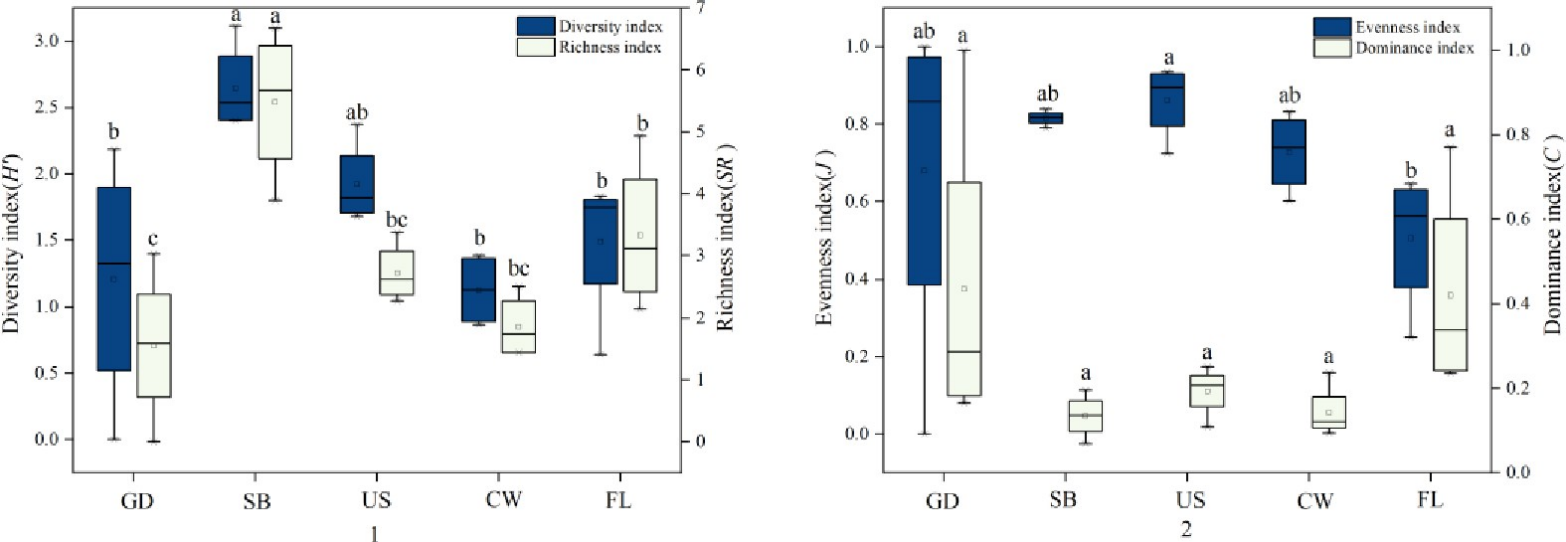
Distribution and change of diversity index (H ’), richness index (SR), evenness index (J) and dominance index (C) of oribatid mites in different moss habitats. Note: Different lowercase letters indicate the significance of distribution difference of diversity index (H ’), richness index (SR), evenness index (J) and dominance index (C) of different habitats; GD: Ground; SB: Surface Shrub; US: Understory; CW: Cave wall; FL: Farmland.

The diversity analysis of the community is used to characterize the complexity of the community composition and evaluate the ecological organization level of the community [37]. The results of community diversity analysis are shown in Fig 5. The diversity index of oribatid mites in shrub was the highest, which was SB>US>FL>GD>CW. Among them, there were significant differences between the ground, cave wall and farmland and shrub (F = 5.271, *df* = 4, p<0.05), but no significant differences between other habitats (F = 5.271, *df* = 4, p>0.05); The richness index of oribatid mites in shrub habitat is the highest, which is SB>FL>US>CW>GD. Among them, there were significant differences between ground, understory, cave wall, farmland and shrub (F = 9.762, *df* = 4, p<0.05), between ground and farmland (F = 9.762, *df* = 4, p<0.05), and no significant differences between other habitats (F = 9.762, *df* = 4, p>0.05); The evenness index understory is the highest, which is US>SB>CW>GD>FL. Among them, only understory and farmland had significant difference (F = 1.446, *df* = 4, p<0.05), and other habitats had no significant difference (F = 1.446, *df* = 4, p<0.05); The dominance index of the ground is the highest, which is in the order of GD>FL>US>CW>SB. There is no significant difference among the five habitats (F = 2.025, *df* = 4, p>0.05).

**Fig 6:**
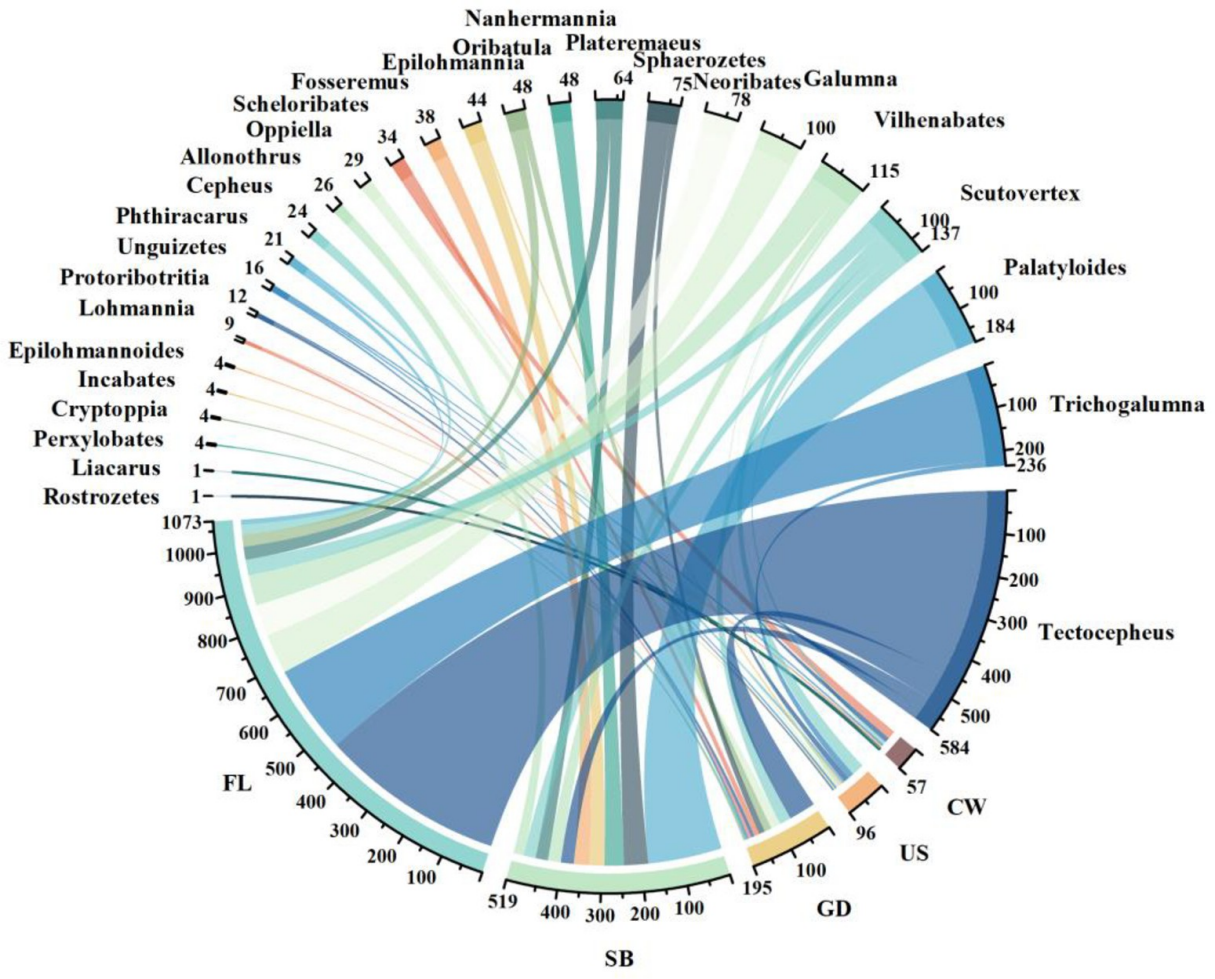
String diagram describing the number of oribatid mites in different moss habitats. Note: The color and the thickness of the line indicate the connection and intensity between different types of oribatid mites and moss habitats. GD: Ground, SB: Shrub, US: Understory, CW: Cave wall, FL: Farmland.

The top ten oribatid mites in different moss habitats in the study area are *Tectocepeus*, *Trichogalumna*, *Palatyloides*, *Scutovertex*, *Vilhenabates*, *Galumna*, *Neoribates*, *Spaerozetes*, *Plateremaeus* and *Nanhermannia* (Fig 6). The result of string diagram shows that different moss environment affects the diversity composition of oribatid mites, and also affects the individual number of oribatid mites in different habitats to a certain extent.

The community similarity analysis is to analyze the main factors affecting the community structure and the important indicators for judging the degree of similarity between communities according to the species composition and quantitative distribution of the community [38]. See Table 3 for the community similarity results of oribatid mites in different moss habitats. The similarity index between ground and cave wall is the highest (0.762), while that between ground and shrub is the lowest (0.125). The similarity index of five habitats is between 0.125 and 0.762. Among them, the ground is very different from the thicket, under forest and farmland; the thicket is very different from the cave wall and between the cave wall and farmland; the undergrowth is medium different from the thicket, cave wall and farmland; the thicket is medium similar to the farmland; the ground is very similar to the cave wall. Among them, the ground is very different from the shrub, the understory is very different from the farmland, the shrub is very different from the cave wall, and the cave wall is very different from the farmland; The understory and shrub are moderately different, and the cave wall and farmland are moderately different; Shrub and farmland are moderately similar; The ground is very similar to the cave wall.

**Table 3:** Similarity of oribatid mite communities in different moss habitats.

Habitat type	GD	SB	US	CW	FL
GD	1	0.125	0.211	0.762	0.216
SB		1	0.349	0.188	0.578
US			1	0.367	0.375
CW				1	0.213
FL					1

Note: GD: Ground, SB: Shrub, US: Understory, CW: Cave wall, FL: Farmland; Community similarity is based on the Jaccard similarity index

**Fig 7:**
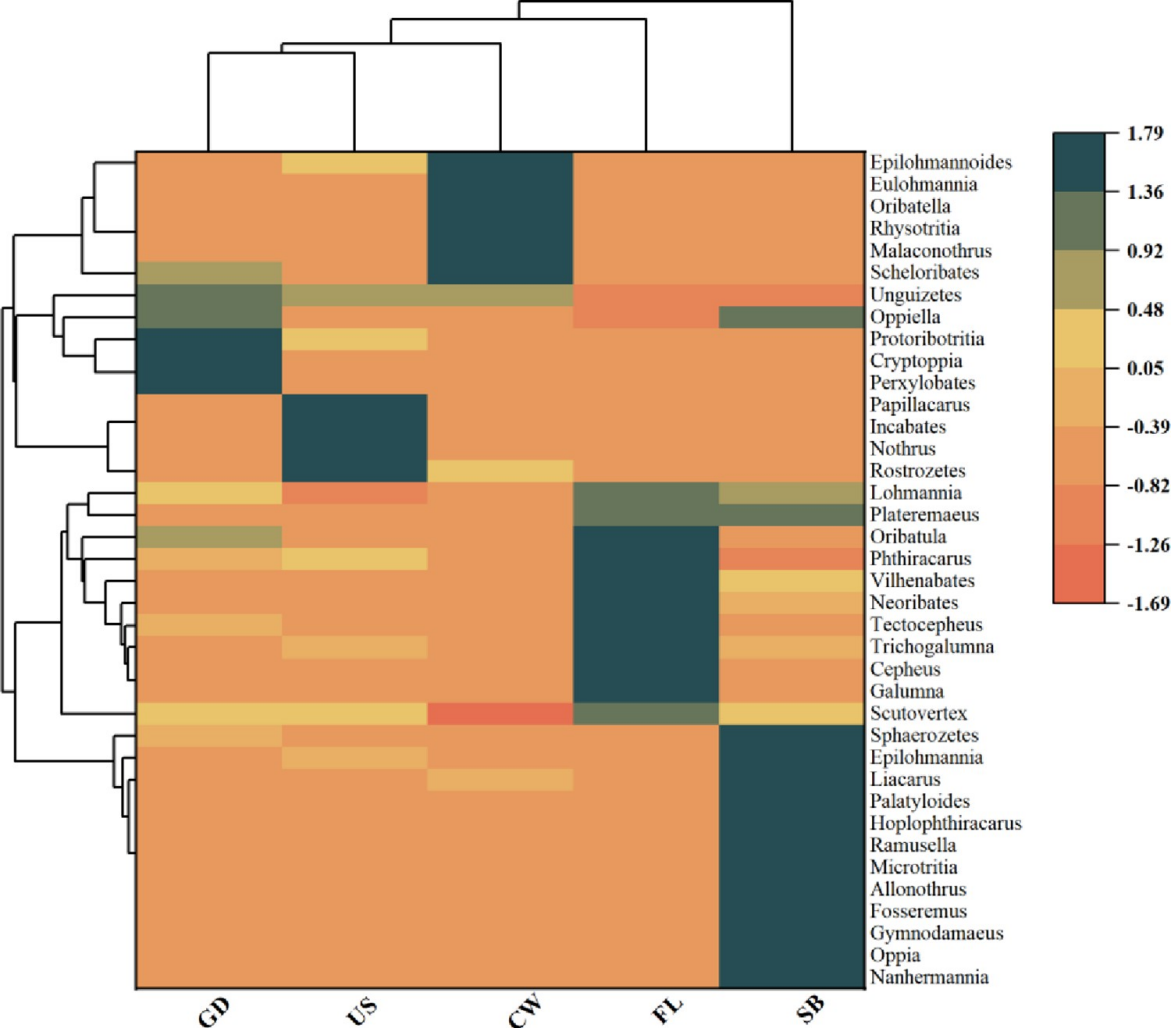
Two-way cluster analysis of oribatid mite community in different moss habitats. Note: GD: Ground, SB: Shrub, US: Understory, CW: Cave wall, FL: Farmland; Standardized the row data and clustered the row and column data evenly, with the clustering type being Euclidean.

In order to further analyze the similarities and differences and similarities of the oribatid mites community among different moss, the oribatid mites with an individual number of more than 1% in each habitat were selected as the original data for cluster analysis. The results showed that the oribatid mites in different moss could be divided into three categories (Fig 7), type 1: GD, US, CW; Type 2: FL; Type 3: SB.

### 3.3 Ecological groups of oribatid mites in different moss habitats

MGP analysis of oribatid mites is a classification method proposed by Aoki to compare the ecological groups of oribatid mites in different environments [36]. MGP analysis can reflect the impact of human activities on the structure of oribatid mites community. The analysis results of ecological groups of oribatid mites in different habitats are shown in Table 4. From the percentage of MGP—Ⅰ group genera, except the ground belongs to G type, shrub, understory, cave wall and farmland belong to O type. From the percentage of MGP—Ⅱ individuals, the ground, shrub and farmland belong to G type, the understory belongs to O type, and the cave wall belongs to GP type. Therefore, based on the results of MGP—Ⅰ and MGP—Ⅱ, the ecological group of the five habitat oribatid mites is dominated by type O.

**Table 4:** Similarity of oribatid mite communities in different moss habitats.

Habitat	Genera percent (%)			Community	Individual percent (%)			Community
Type	Macropylina	Gymnona	Poronota	Types	Macropylina	Gymnona	Poronota	Types
GD	8.82±8.82	67.55±16.19	23.63±9.01	G	2.81±2.81	75.1±11.28	22.09±8.91	G
SB	28.22±1.33	46.41±2.62	25.37±2.08	O	16.54±1.56	59.78±5.6	23.68±4.2	G
US	33.8±14.36	21.43±9.14	44.77±5.77	O	30.52±16.6	22.6±14.04	46.88±11.12	O
CW	26.79±15.53	34.64±11.8	38.57±10.83	O	10.75±6.51	42.47±14.97	46.78±12.44	GP
FL	24.03±11.06	37.44±8.06	38.53±8.12	O	7.1±3.97	56.62±19.03	36.28±20.21	G

Note: GD: Ground, SB: Shrub, US: Understory, CW: Cave wall, FL: Farmland; The values in the table are mean and standard deviation.

### 3.4 Trophic structure of oribatid mites

Schneider divided the mites into four major groups based on their trophic structure, including predators, carnivores, scavengers, omnivores, secondary consumers, primary consumers, planters, and fungal eaters [18]. There are a total of 72 genera of oribatid mites in different moss habitats in the study area. Based on relevant literature and books, 42 known genera were classified according to the above groups. The results are shown in Table 5. The results showed that in different moss habitats, different groups of oribatid mites were distributed in each Trophic level, and these 42 species of oribatid mites initially constituted the relatively complete decomposition functional group or Decomposer trophic structure of the bryophyte ecosystem in the study area.

**Table 5:** Trophic structure of oribatid mites.

Feeding Guild	Oribatid Mite Groups	Food Materials	Source
Carnivores/Scavengers/Omnivores	*Oribotritia*	Living and dead animals (nematodes, collembolans) and fungi	Book [32]
	*Vepracarus*		Book [32]
	*Hypochthonius*		literature [39]
	*Rostrozetes*		literature [12]
	*Lohmannia*		literature [12]
	*Trhypochthonius*		literature [39]
	*Lasiobelba*		literature [40]
	*Arcoppia*		literature [40]
	*Oxyoppia*		literature [41]
	*Condyloppia*		literature [41]
	*Ramusella*		literature [41]
	*Oppia*		literature [39]
	*Oppiella*		literature [39]
	*Cryptoppia*		literature [41]
	*Galumna*		literature [39]
	*Trichogalumna*		literature [40]
	*Nothrus*		literature [39]
	*Protokalumna*		literature [40]
Secondary decomosers	*Archoplophora*	Predominantly fungi, in part litter	Book [32]
	*Epilohmannoides*		Book [32]
	*Epilohmannia*		Book [32]
	*Phthiracarus*		Book [32]
	*Eupterotegaeus*		Book [32]
	*Camisia*		Book [32]
	*Mixacarus*		Book [32]
	*Malaconothrus*		literature [18]
	*Damaeus*		Book [32]
	*Cepheus*		literature [18]
	*Nanhermannia*		literature [18]
	*Carabodes*		literature [18]
	*Scheloribates*		literature [39]
	*Chamobatidae*		literature [40]
Primary decomposers/Fungivores	*Eulohmannia*	Predominantly litter	Book [32]
	*Liacarus*		literature [18]
	*Tectocepheus*		literature [39]
	*Oribatella*		literature [18]
	*Oribatula*		literature [18]
Phycophages/ Fungivores	*Zetorchestes*	Lichens and algae	literature [39]
	*Cultroribula*		literature [39]
	*Mochlozetes*		literature [39]
	*Podoribates*		literature [39]
	*Melanozetes*		literature [42]

## 4 Discussion

### 4.1 The composition and distribution of oribatid mite community in different moss habitats show differences

As a typical pioneer plant, moss is a very suitable model for ecological research on habitats with its attached rocks or epiphytic covers. Relatively simple micro arthropod communities live in moss and even provide food for some animals [26]. A total of 2352 oribatid mites belonging to 45 families, 72 genera were captured in 5 moss habitats in the study area, and most of them were Gymnonota. There are obvious differences in the number of families, genera, individuals and individual density of oribatid mites in the five moss habitats. The number of group and individuals of oribatid mites in shrub and farmland is much higher than that understory, on the ground and in cave wall, which is consistent with the conclusion of Stanislam and Julio and other scholars that there are more mites in shrub moss [14, 43–46]. There are differences in micro habitats in different moss habitats, which directly affect the diversity and species composition of mite communities [44]. The reason for the difference in species diversity of oribatid mites in the study area may be that the shrub and farmland have well developed vegetation, and a large number of dead branches and leaves exist in the moss. The humus layer is thick, and the food sources of oribatid mites are diverse. Therefore, the most abundant oribatid mites exist in the shrub and farmland [47]. However, the lack of vegetation on the ground and on the cave wall leads to the lack of stable food sources for the oribatid mites. Therefore, the oribatid mites in these two habitats have fewer groups and numbers.

Mites often inhabit moss, soil, litter, grassland and lichen, and have the characteristics of high sensitivity, strong tolerance, wide distribution and mixed feeding habits, as well as the sensitive function of responding to ecosystem and indicating environmental quality changes [48, 49]. They play an important role in nutrient cycling and environmental indicators in the ecological environment [50, 51]. The composition of dominant genera of oribatid mites in different moss habitats in the study area is different. In ground moss, the dominant genera of oribatid mites are *Oppiella*, *Tectocepeus* and *Scutovertex*; In shrub moss, the dominant genus of oribatid mites is *Palatyloides*; In understory mosses, the dominant genera of oribatid mites are *Tectocepeus*, *Scutovertex* and *Trichogalumna*; In cave wall mosses, the dominant genera of oribatid mites are *Tectocepeus*, *Scutovertex* and *Schelaribates*; The dominant genera of oribatid mites in farmland mosses are *Tectocepheus* and *Trichogaluna*. It can be seen that with the change of moss habitat, the dominant genera of oribatid mites show different distribution characteristics, and the individual numbers of *Tectocepheus* and *Trichogaluna* occupy the main position in the dominant genera of oribatid mites, which can be used as the indicator species of the moss environment in the cave. International research results show that *Tectocepheus* is abundant in roof moss, forest ground moss and arctic tundra [45, 46, 52]. Research on mites in moss habitats of different rocky desertification grades in karst regions of southwest China shows that *Trichogaluna* and *Nanorchestes* are dominant genera, and *Trichogaluna* also exists in soil environment in karst regions [53], the results indicated that the dominant genus composition of mites in the moss in this paper was basically consistent with the conclusion of most papers on mites, but there were some differences, which might be caused by the difference of environmental factors between habitats, such as different vegetation types, rock temperature, air temperature and humidity, solar illumination and so on in the moss microhabitat [14].

### 4.2 Diversity and similarity of oribatid mite communities in different moss habitats show differences

Higher community diversity index and richness index indicate that there is a longer food chain and more complex food web in the ecosystem, thus enhancing the stability of the community [54, 55]. The diversity index of mites in different moss habitats in the study area is generally higher than that of mites in rose mixed agricultural forest, burning agricultural land and rosa roxburghii land in karst area [56–58], indicating that the ecological environment of mites in cave moss habitats is more stable than that of human disturbance. A lot of research shows that the diversity of mite community is related to the composition of vegetation components and the diversity of covering plants, and the quantity and nature of its litter may have a decisive impact on mite community [24, 59]. In this paper, the diversity index and richness index of shrub, farmland and understory are ahead of the ground and cave wall, and these three habitats have more abundant vegetation and litter than the ground and cave wall, which also proves the findings that their community diversity index is higher and the mite community structure is more stable. *Tectocepheus*, *Trichogalumna*, *Palatyloides* and other top ten oribatid mites in the study area are distributed in different moss environments to varying degrees, indicating that the diversity composition and individual number of oribatid mites are affected to some extent by the differences of moss habitats.

As an important indicator to measure the relationship between different habitats and soil mite community structure, community similarity analysis plays an important role in community structure analysis [60], and is also an important indicator to characterize the relationship between different habitat types and mite communities. According to the Jaccard similarity index of soil mites in the study area, the similarity index between ground and cave wall is the highest, and the similarity index between ground and shrub is the lowest. The similarity index of five habitats ranges from 0.125 to 0.762. Except that the similarity between ground and cave wall, shrub and farmland is high, the similarity between other habitats is low. Cluster analysis of oribatid mites in different moss habitats showed that the ground, cave wall and understory belong to the same category, the shrub is a separate category, and the farmland is a separate category. Relevant studies also show that the ecological environment has a significant impact on the community structure. The more similar the ecological environment is, the more similar the biological communities will be. Manh’s survey of mites in different habitats in Vietnam shows that the habitat type has a great impact on the species similarity index [61]. Jiao et al.’s research on soil mites in the forest on the north slope of Laotudingzi, Liaoning Province shows that the more similar the vegetation is, the more similar the soil mites are [62], M A. Minor and Xia also believed that the community similarity was closely related to the vegetation composition of the habitat [63, 64]. The lack of vegetation on the ground and cave wall, and the abundance of vegetation in shrub and farmland in the study area may cause the difference in the similarity index of oribatid mites in these moss habitats.

### 4.3 The ecological group of oribatid mites in different moss habitats is dominated by type O

Due to its rich morphology and ecological form, oribatid mites can play a geochemical role and participate in the turnover of nutrients in the ecological environment [65]. MGP analysis of oribatid mites is a classification method proposed by Aoki to compare the ecological groups of oribatid mites communities in different environments [36]. MGP analysis can reflect the impact of human interference on the structure of oribatid mite communities. The areas with good ecological environment usually show O and P types. Therefore, oribatid mites are an ideal biological indicator species for monitoring the ecological environment. MGP—Ⅰ of oribatid mites in 5 habitats in the study area is dominated by type O, and MGP—Ⅱ of oribatid mites is dominated by type G and type O. In terms of quantitative structure, the ground and farmland are of type G, which is consistent with the conclusion of Zhang and Wu [66, 67], mainly related to the ground and agricultural land with frequent human activities. Wu et al. also pointed out that the number of low-level oribatid mites (M group) is relatively small in areas with more human activities, and the number structure type of oribatid mites is often G or GP [67]. However, according to the percentage of genera of MGP—Ⅰ and the percentage of individuals of MGP—Ⅱ, the ecological group of oribatid mites in different moss habitats in the study area is dominated by type O, which is similar to the research results of Shibing Karst Forest, Fanjing Mountain, rocky desertification area management area and Chishui Alsophila spinulosa Nature Reserve [42, 47, 66, 68], indicating that the comprehensive environmental performance of different moss habitats is good.

### 4.4 33 species of oribatid mites preliminarily constitute the nutrient structure of cave moss habitat

Oribatid mites are very suitable as indicators of ecological environment, because they exist in most terrestrial habitats, have high diversity and abundance, are easy to sample, show good adaptability to multiple environments, and have low spatial mobility [12]. Oribatid mites play an important role in nutrient cycling and humus formation of ecosystems [32], and also play an indispensable role in litter decomposition and nutrient cycling [16]. A better understanding of the trophic structure and decomposition function group of oribatid mites is not only helpful to understand the species diversity of oribatid mites in the study area, but also of positive significance to understand the functions of these oribatid mites in the process of organic matter decomposition [22]. In five different bryophyte habitats, different groups of oribatid mites are distributed in each Trophic level. A total of 42 species of oribatid mites constitute a relatively complete decomposition functional group or Decomposer trophic structure of the bryophyte ecosystem in the study area. The bryophytes under different habitat types provide different Ecological niche for oribatid mites, which leads to the differences and differences of oribatid mite groups in bryophytes in different habitats. This Ecological niche may be related to the trophic needs of oribatid mites. Due to the complexity of the relationship between the Karst cave environment and the community structure of oribatid mites in mosses, the research shows that *Tectocepheus* is the dominant group in the ground and surface shrub habitats, *Oppiella* is the dominant group in the ground habitats, *Trichogalumna* is the dominant group in the surface shrub habitats, and *Scheloribates* is the dominant group in the cave wall habitats. Among them, *Tectocepheus* plays the role of primary decomposer, and often exists in the environment of early succession and less interference [69], which may be related to less human interference on the Karst Plateau cave ground and more humus in the forest habitat; *Oppiella* and *Trichogalumna* have stable food sources in different habitats, so they have certain characteristics of surface aggregation; *Scheloribates* mainly feeds on fungi, which may be due to the distribution of more lichens and mosses near cave wall habitats. In order to meet the trophic, reproductive and physiological needs of different Oribatid mites in moss, their community structure characteristics are constantly adjusted with the changes of habitats. Therefore, the indication of cave environment by cave Oribatid mites requires a series of comprehensive studies such as biological group analysis.

## 5 Conclusion

The group and quantity of oribatid mites in different moss habitats in the cave are relatively rich, and the dominant group of oribatid mites represented by *Tectocepheus* and *Trichogalumna* can be used as a biological indicator of the moss habitat in the cave. The community diversity index and community similarity index of oribatid mites in different moss habitats changed significantly. The ecological group of oribatid mites in the study area is dominated by type O, and 42 species of oribatid mites constitute a relatively complete functional group or nutrient structure of decomposers in the moss ecosystem in the study area. This study enriches the study of mites in karst cave mosses, and provides theoretical significance for the protection of cave biodiversity in karst areas. The next work on the adaptation mechanism of oribatid mites in cave mosses to environmental factors needs to be carried out.

## Supporting information

S1 Appendix (DOCX)
