# Supplementary material for: Differences of oribatid mite community and trophic structure between karst caves and surface different moss habitats
Source: PLoS One. 2023 Aug 17;18(8):e0290144. doi: 10.1371/journal.pone.0290144 (PMC10434907; doi:10.1371/journal.pone.0290144)
Supplement: S1 Appendix — (DOCX) [file pone.0290144.s001.docx]

**Appendix**

Table 3. Community composition and distribution of oribatid mites in different moss habitats

| Family | Genus | Different habitat types | | | | |  |
| --- | --- | --- | --- | --- | --- | --- | --- |
|  |  | GD | SB | US | CW | FL | Total |
|  |  | Ind.(Dom.) | Ind.(Dom.) | Ind.(Dom.) | Ind.(Dom.) | Ind.(Dom.) |  |
| Mesoplophoridae | *Archoplophora* |  | 4（+） |  |  |  | 4（+） |
| Hypochthoniidae | *Hypochthonius* | 1（+） | 4（+） |  |  | 3（+） | 8（+） |
| Lohmanniidae | *Mixacarus* |  | 2（+） |  |  |  | 2（+） |
|  | *Lepidacarus* |  | 1（+） | 1（+） |  |  | 2（+） |
|  | *Lohmannia* | 7（++） | 9（++） |  | 2（++） | 12（++） | 30（++） |
|  | *Vepracarus* | 1（+） | 1（+） |  |  |  | 2（+） |
|  | *Papillacarus* |  |  | 3（++） |  |  | 3（+） |
| Eulohmanniidae | *Eulohmannia* |  |  |  | 1（++） |  | 1（+） |
| Epilohmanniidae | *Epilohmannoides* |  | 2（+） | 2（++） | 4（++） | 7（+） | 15（+） |
|  | *Epilohmannia* |  | 38（++） | 6（++） |  | 3（+） | 47（++） |
| Oribotritiidae | *Oribotritia* | 1（+） |  |  |  |  | 1（+） |
|  | *Protoribotritia* | 8（++） | 5（+） | 4（++） | 1（++） | 1（+） | 19（+） |
| Euphthiracaridae | *Rhysotritia* |  | 1（+） |  | 1（++） | 1（+） | 3（+） |
|  | *Microtritia* |  | 8（++） |  |  |  | 8（+） |
| Synichotritiidae | *Synichotritia* |  | 2（+） | 1（+） |  |  | 3（+） |
| Phthiracaridae | *Phthiracarus* | 4（++） | 3（+） | 7（++） | 1（++） | 13（++） | 28（++） |
|  | *Hoplophorella* |  | 3（+） |  |  |  | 3（+） |
|  | *Hoplophthiracarus* |  | 18（++） |  |  | 2（+） | 20（+） |
| Camisiidae | *Camisia* |  | 3（+） |  |  | 3（+） | 6（+） |
| Nothridae | *Nothrus* |  | 1（+） | 2（++） |  | 7（+） | 10（+） |
| Pterochthoniidae | *Pterochthonius* |  |  |  |  | 1（+） | 1（+） |
| Trhypochthoniidae | *Trhypochthonius* |  | 3（+） |  |  | 5（+） | 8（+） |
|  | *Allonothrus* |  | 26（++） | 1（+） |  | 3（+） | 30（++） |
| Malaconothridae | *Malaconothrus* |  |  |  | 1（++） |  | 1（+） |
| Nanhermanniidae | *Nanhermannia* |  | 48（++） |  |  | 10（+） | 58（++） |
| Hermanniidae | *Phyllhermannia* |  | 1（+） | 1（+） |  | 5（+） | 7（+） |
| Plasmobatidae | *Plasmobates* |  | 2（+） |  |  |  | 2（+） |
| Neoliodidae | *Palatyloides* |  | 184（+++） |  |  | 2（+） | 186（++） |
| Gymnodamaeidae | *Gymnodamaeus* |  | 14（++） |  |  | 5（+） | 19（+） |
| Plateremaeidae | *Plateremaeus* |  | 31（++） |  |  | 33（++） | 64（++） |
| Damaeidae | *Damaeus* |  | 4（+） |  |  |  | 4（+） |
| Damaeolidae | *Fosseremus* |  | 38（++） |  |  |  | 38（++） |
| Eremaeidae | *Eremaeus* |  | 3（+） |  |  | 1（+） | 4（+） |
| Cepheidae | *Cepheus* | 2（+） | 1（+） |  |  | 24（++） | 27（++） |
|  | *Eupterotegaeus* | 2（+） | 3（+） |  |  |  | 5（+） |
| Zetorchestidae | *Zetorchestes* |  | 7（+） |  |  | 8（+） | 15（+） |
| Liacaridae | *Liacarus* |  | 12（++） |  | 1（++） |  | 13（+） |
| Astegistidae | *Cultroribula* |  | 2（+） |  |  |  | 2（+） |
| Carabodidae | *Carabodes* |  | 1（+） |  |  |  | 1（+） |
|  | *Yoshibodes* |  | 1（+） | 1（+） |  | 1（+） | 3（+） |
| Otocepheidae | *Dolicheremaeus* | 1（+） | 3（+） |  |  |  | 4（+） |
| Suctobelbidae | *Allosuctobelba* |  | 1（+） |  |  |  | 1（+） |
| Oppiidae | *Oppia* |  | 19（++） |  |  | 2（+） | 21（+） |
|  | *Lasiobelba* |  |  |  |  | 1（+） | 1（+） |
|  | *Arcoppia* | 1（+） |  |  |  |  | 1（+） |
|  | *Oxyoppia* |  | 7（+） |  |  | 1（+） | 8（+） |
|  | *Condyloppia* |  | 1（+） |  |  |  | 1（+） |
|  | *Oppiella* | 24（+++） | 21（++） | 5（++） | 1（++） | 3（+） | 54（++） |
|  | *Cryptoppia* | 4（++） | 3（+） |  |  |  | 7（+） |
|  | *Ramusella* |  | 16（++） | 1（+） |  | 3（+） | 20（+） |
| Tectocepheidae | *Tectocepheus* | 67（+++） | 32（++） | 14（+++） | 12（+++） | 459（+++） | 584（+++） |
| Scutoverticidae | *Scutovertex* | 29（+++） | 30（++） | 31（+++） | 8（+++） | 39（++） | 137（++） |
| Oribatellidae | *Oribatella* |  |  | 1（+） | 1（++） |  | 2（+） |
| Parakalummidae | *Neoribates* |  | 14（++） |  |  | 78（++） | 92（++） |
| Oribatulidae | *Oribatula* | 18（++） | 5（+） |  |  | 30（++） | 53（++） |
|  | *Incabates* |  | 1（+） | 4（++） |  |  | 5（+） |
| Oripodidae | *Parapirnodus* |  | 4（+） |  |  | 5（+） | 9（+） |
| Scheloribatidae | *Scheloribates* | 13（++） |  | 2（++） | 21（+++） | 4（+） | 40（++） |
| Mochlozetidae | *Mochlozetes* |  | 4（+） |  |  |  | 4（+） |
|  | *Podoribates* |  | 1（+） | 1（+） |  | 1（+） | 3（+） |
| Haplozetidae | *Rostrozetes* | 2（+） | 3（+） | 3（++） | 1（++） |  | 9（+） |
|  | *Perxylobates* | 4（++） |  |  |  |  | 4（+） |
|  | *Vilhenabates* |  | 32（++） | 5（++） | 2（++） | 76（++） | 115（++） |
|  | *Peloribates* |  | 2（+） |  |  |  | 2（+） |
| Xylobatidae | *Xylobates* | 1（+） |  |  |  |  | 1（+） |
| Ceratozetidae | *Melanozetes* |  | 5（+） | 1（+） |  | 3（+） | 9（+） |
|  | *Sphaerozetes* | 15（++） | 60（++） |  |  | 5（+） | 80（++） |
| Chamobatidae | *Chamobates* |  | 1（+） | 1（+） |  | 2（+） | 4（+） |
| Onychobatidae | *Unguizetes* | 6（++） | 1（+） | 5（++） | 5（++） |  | 17（+） |
| Galumnidae | *Galumna* |  |  |  |  | 100（++） | 100（++） |
|  | *Trichogalumna* |  | 19（++） | 15（+++） |  | 221（+++） | 255（+++） |
|  | *Protokalumna* |  | 2（+） |  |  | 4（+） | 6（+） |
| Number of groups |  | 21 | 60 | 25 | 16 | 41 | 72 |
| Number of individuals |  | 211 | 773 | 118 | 63 | 1187 | 2352 |

Note: GD: Ground; SB: Surface Shrub; US: Understory; CW: Cave wall; FL: Farmland; Ind: Individual quantity; Dom: Dominance; Individual number accounts for more than 10% of the total catch are marked as dominant groups (+++), those whose proportion is 1% - 10% are marked as common groups (++), and those whose proportion is less than 1% are rare groups (+).
